# Immune Modulation and Immune-Mediated Pathogenesis of Emerging Tickborne Banyangviruses

**DOI:** 10.3390/vaccines7040125

**Published:** 2019-09-20

**Authors:** Crystal A. Mendoza, Hideki Ebihara, Satoko Yamaoka

**Affiliations:** 1 Mayo Clinic Graduate School of Biomedical Sciences, Mayo Clinic, Rochester, MN 55905, USA; Mendoza.Crystal@mayo.edu; 2 Department of Molecular Medicine, Mayo Clinic, Rochester, MN 55905, USA

**Keywords:** heartland, HRTV, severe fever with thrombocytopenia syndrome virus, SFTSV, SFTS, Banyangvirus, Guertu virus, interferon antagonism

## Abstract

In the last decade, the emergence of several, novel tickborne viruses have caused significant disease in humans. Of interest are the tickborne banyangviruses: Severe fever with thrombocytopenia syndrome virus (SFTSV), Heartland virus (HRTV), and Guertu virus (GTV). SFTSV and HRTV infection in humans cause viral hemorrhagic fever-like disease leading to mortality rates ranging from 6–30% of the cases. The systemic inflammatory response syndrome (SIRS) associated with SFTSV infection is hypothesized to contribute significantly to pathology seen in patients. Despite the severe disease caused by HRTV and SFTSV, there are no approved therapeutics or vaccines. Investigation of the immune response during and following infection is critical to the generation of fully protective vaccines and/or supportive treatments, and overall understanding of viral immune evasion mechanisms may aid in the development of a new class of therapeutics.

## 1. Introduction

Tickborne banyangviruses, known causing viral hemorrhagic fever (VHF)-like disease in humans, are a recent emerging threat to human health worldwide. Taxonomically, tickborne Banyangvirus belong to the genus *Banyangvirus* in the family *Phenuiviridae* of the order *Bunyavirales*. The genus *Banyangvirus* consists of three species: *Huiayangshan banyangvirus*, *Heartland banyangvirus*, and *Guertu banyangvirus*, which are represented by SFTSV, HRTV, and GTV, respectively [1]. In this review, we discuss in detail the innate/adaptive immune responses and pro-inflammatory response, and their respective roles in pathogenesis during tickborne banyangvirus infection as it relates to disease severity and patient outcome.

## 2. Tick-Borne Banyangviruses

### 2.1. Severe Fever with Thrombocytopenia Syndrome Virus (SFTSV)

SFTSV was identified in 2009 in the Hubei and Henan provinces in eastern China when several patients presented with febrile illness similar to anaplasmosis, a tickborne rickettsial disease [2]. Following its isolation and initial characterization, human cases of SFTSV were subsequently reported in South Korea [3,4,5], and Japan [6]. Most recently, samples from patients in Vietnam suggested endemic SFTSV [7]. Importantly, there were 5360 cases of SFTSV from 2011–2016 in China with 343 fatalities (6.4% case fatality rate) [8]. Between 2013–2015, there were 172 reported cases of SFTSV in South Korea including 56 fatal cases (32.5% case fatality rate) [9]. From March 4, 2013 through March 31, 2019, 394 cases of SFTSV were reported in Japan with an estimated case fatality rate of 27% [10]. The predominant vector of SFTSV is the Asian long-horned tick, *Haemaphysalis longicornis* among other tick species, which transmit SFTSV [3,4,11,12,13]. Epidemiological sampling of mammalian hosts, such as sheep, cattle, dogs, goats, pigs, cheetahs in zoos, and chickens and tick vectors have further established endemic SFTSV in China, South Korea, and Japan [14,15,16,17]. In addition to transmission from the *H. longicornis* vector, and other tick vectors, there have been cases of human-to-human transmission of SFTSV via contact with blood and bodily fluids, including nosocomial settings [15,18,19,20,21,22,23], and potential of SFTSV transmission from companion animals to humans poses a risk to pet owners as well as veterinary professionals [15,24]. Because of the pathogenic potential and ability of SFTSV to cause nosocomial human-to-human transmission, SFTSV is classified as a Category C Priority Pathogen by National Institutes of Health (NIH) and included on the List of Blueprint priority diseases by World Health Organization (WHO).

### 2.2. Heartland Virus (HRTV)

In the United States, HRTV was first identified in Missouri in two male patients who reported tick bites followed by fever, fatigue, and headache [25]. HRTV shares 73% and 63% sequence identity to the RNA-dependent RNA polymerase protein and the nucleoprotein of SFTSV, respectively [25]. The vector for HRTV has been identified as the Lone Star tick, *Amblyomma americanum*, through sampling of captured nymph and adult ticks as well as studies using experimentally infected ticks [26,27]. Wildlife sampling studies of mammals, such as raccoons, white-tailed deer, horses, coyotes, and moose, in the geographic range of *A. americanum* have shown neutralizing antibodies to HRTV in 13 states [28]. As of this writing, 40 cases of HRTV disease have been reported to the Centers for Disease Control and Prevention (CDC) with three fatalities [29]. Some reports estimated the seroprevalence of HRTV neutralizing antibodies from blood donor samples to be 0.9% in northwestern Missouri, U.S. suggesting the potential for HRTV disease to remain undetected in populations [30]. The disease severity, and pathogenic potential for HRTV has led to its classification as a BSL3 pathogen and as a Category C Priority Pathogen by the NIH.

### 2.3. Guertu Virus (GTV)

The identification and isolation of GTV in Xinjiang province in China from *Dercamentor muttalli* ticks was reported in 2018 [31]. Sequencing of GTV from pooled tick samples revealed 77–86% amino acid similarity to SFTSV [31]. Human and animal-derived cell lines showed susceptibility to GTV similar to SFTSV [31]. While limited studies have been conducted on GTV due to its recent identification, serological evidence of herdsmen and farmers supports endemic GTV infection in humans [31]. Importantly, most recently, one laboratory-confirmed case of human SFTSV infection was reported in the Xinjiang province [32]; ticks sampled from the Xinjiang province were also shown to be SFTSV positive. This indicates an overlapped distribution of SFTSV and GTV in Xinjiang province. Since SFTSV and GTV are serologically cross-reactive, future laboratory tests should have high specificity in order to distinguish between SFTSV and GTV that prevents potential misdiagnosis of infected patients.

## 3. Basic Virology of Banyangviruses

### 3.1. Genome Organization

**Figure 1:**
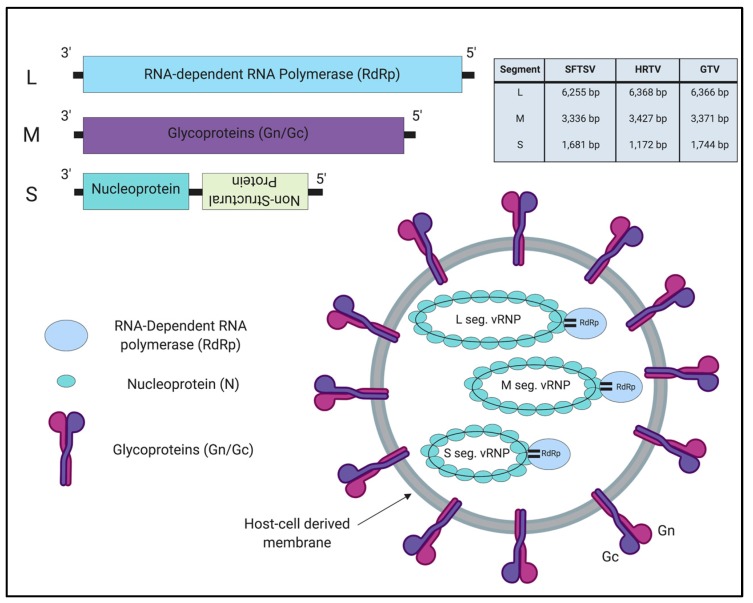
Genome organization and viral particle of tickborne banyangviruses. The tripartite segmented genome is comprised of three segments: Large (L), Medium (M), and Small (S). The L segment encodes the viral RNA-dependent RNA polymerase. The M segment encodes two glycoproteins, Gn and Gc. The S segment is encoded in ambisense orientation and encodes the viral nucleoprotein (N) and the non-structural protein (NSs), which is encoded in the opposite orientation to the N protein and therefore is written in upside down text. Viral particles measure 80-100 nm in diameter for tickborne banyangviruses. The length of each segment for SFTSV, HRTV, and GTV is noted in the table.

SFTSV, HRTV, and GTV belong to the *Banyangvirus* genus in the *Phenuiviridae* family and order *Bunyavirales* [33]. Viruses in this family contain a segmented, tripartite genome: Large (L), Medium (M), and Small (S) segments; the L and M segments are encoded in the negative-sense orientation, while the S segment is in ambisense orientation (Figure 1) [34]. The L segment encodes the RNA-dependent RNA polymerase (RdRp) which mediates transcription and replication of the viral genome. The M segment encodes a polyprotein precursor that is cleaved into two glycoproteins, Gn and Gc, which mediate viral entry into cells, and incorporation of the nucleocapsid/ribonucleoprotein complex into the viral particle [35]. Finally, the S segment encodes the nucleocapsid protein (N) and the non-structural protein (NSs). The N protein encapsidates the viral genome RNA, and is essential for genome transcription/replication [36]. The NSs protein, an interferon (IFN) antagonist, has been widely studied in the field due to its functional similarity to the NSs proteins of viruses in the *Phenuiviriade* family [37,38]. The IFN-antagonistic function of NSs proteins from SFTSV, HRTV, and GTV are discussed in detail in Section 4 below.

### 3.2. Target Tissues/Cells and Cellular Receptors

Autopsies from patients with SFTS and HRTV disease have shown viral antigen present in infiltrating lymphocytes in various tissues, including but not limited to spleen, kidneys, lungs and heart [39,40,41,42]. In particular, specific studies have shown HRTV antigen positive macrophages and Kupffer cells in the liver of a patient with HRTV-associated death [43]. Moreover, autopsy studies from SFTS-associated deaths have shown SFTSV antigen-positive lymphoid and hematopoietic cells in lymph nodes, palatine tonsils, and other organs [39].

The cellular receptor for SFTSV and HRTV has been identified as dendritic cell-specific intercellular adhesion molecule-3-grabbing non-integrin (DC-SIGN), which is expressed on macrophages and dendritic cells, implying that these cells are the primary cellular target for viral replication [44,45]. Indeed, in vitro studies have demonstrated the ability of immortalized human and mouse macrophage cell lines to support replication of SFTSV [46,47,48]. Moreover, other C-type lectins such as DC-SIGN related (DC-SIGNR) and liver and lymph node sinusoidal endothelial cell C-type lectin (LSECtin), when expressed on non-permissive cells, can support viral entry [49]. One report, however, implicated the involvement of non-muscle myosin heavy chain IIA in SFTSV entry in several mammalian cell lines [50]. Further studies are required to establish the relevance of this receptor as well as DC-SIGN during pathogenesis.

### 3.3. Clinical Disease Course

**Figure 2:**
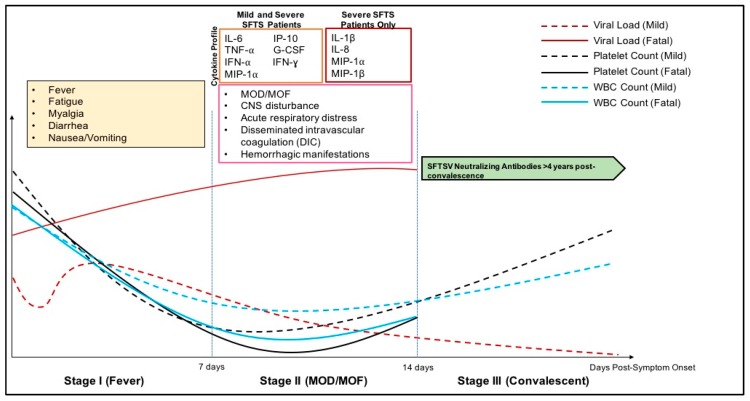
Clinical disease progression of SFTSV and HRTV. Clinical disease progression of HRTV and SFTSV infection is described above including laboratory parameters, and symptoms seen in both mild and severe patients. Induction of inflammatory cytokines is seen during the acute phase of infection, and returns to normal levels in mild patients. MOF: multi-organ failure; MOD: multi-organ dysfunction.

The clinical disease courses for SFTS and HRTV disease has been well-described by several groups, which is characterized by three phases: Stage I (fever stage), Stage II (multiorgan dysfunction (MOD)/multiorgan failure (MOF) stage), and Stage III (convalescent stage) (Figure 2) [42,51,52,53]. The fever stage follows a 3–7-day incubation period after a suspected tick bite, exposure to SFTS patients (nosocomial transmission, bodily fluid contact), or contact with an SFTSV-infected animals [42]. For HRTV, while the incubation period from exposure to symptom onset is not well-known, patients have reported 14 days from suspected tick bite to symptom onset [29]. In Phase I, patients infected with SFTSV and HRTV initially experience fever, diarrhea, myalgia, and headache. Laboratory parameters of infected patients have been reported as: thrombocytopenia, leukopenia, and elevated aspartate amino transferase (AST) and alanine aminotransferase (ALT) levels [52]. In the case of SFTS, further clinical evaluation has also shown elevated pro-inflammatory cytokine and chemokine levels in patients correlated to severe disease further discussed in Section 4. Within the first week post-symptom onset, thrombocytopenia, leukopenia, and other clinical indicators return to normal levels in mild SFTS patients while viral titers decline out to 14 days post initial symptoms onset. Following the first clinical stage, severe SFTS patients reach the MOD/MOF stage, which is characterized clinically by elevated AST/ALT, and high viral titer reaching 1 × 10^8^ viral copies/mL and can experience secondary bacterial or fungal infections, hemorrhagic manifestations, central nervous system (CNS) disorders, disseminated intravascular coagulation, shock, and acute respiratory distress [39,42,51,54,55].

### 3.4. Pathology

Gross pathological findings in both HRTV and SFTS patients have found hemorrhage in the gastrointestinal tract, abdomen and lungs, liver necrosis, and lymphadenopathy closest to tick bite region [39,40,43,56,57,58,59]. Indeed, there is severe necrosis and infiltration of lymphocytes, histocytes, atypical lymphoid cells, and abundant nuclear debris in the lymph node closest to the region of the tick bite while other lymph nodes did not show necrotizing lymphadenitis [6,39,56,58]. Upon autopsy, the lungs of deceased SFTS patients have shown pulmonary edema in addition to hemorrhage, infarction, and diffuse alveolar damage indicating acute lung injury [39,56]. The liver of SFTS and HRTV patients showed single cell necrosis and mild periportal lymphocytic infiltration [39,43,56,57]. Examination of the heart indicated pericardial fluid retention and vacuolar degradation of the pericardium likely caused by SFTSV infection as the patient had been previously healthy with no reported cardiac issues [6]. Indeed, a separate case of an SFTS patient who also had no previous cardiac issues was reported to have fulminant myocarditis caused by SFTSV infection [60]. Immunohistochemical staining for SFTSV or HRTV antigen showed antigen (N protein) positive cells in atypical lymphoid cells of systemic lymph nodes and the spleen of both SFTS or HRTV disease patients [40,56,57,58,59,61]. Studies of parenchymal cells of various organs from SFTS patients did not show SFTSV antigen present [39,56,57,58,61]. Furthermore, bone marrow aspirates of SFTS and HRTV disease patients also demonstrated hemophagocytic lymphohistiocytosis (HLH) secondary to viral infection [6,43,62]. The engulfment of blood cells, including erythrocytes and leukocytes by macrophages is apparent in hematoxylin and eosin (H&E) staining from bone marrow aspirates in both severe and non-severe SFTS and HRTV disease patients [6]. The degree of HLH is likely correlated with severity of disease, since hemophagocytosis causes thrombocytopenia and lymphopenia due to the engulfment of platelets and lymphocytes, respectively. Several reports also suggested the involvement of HLH in the CNS disorder seen in SFTS patients [39,40,43,56,58,59,61,62]. It has been known that the over-production of pro-inflammatory cytokines resulting in systemic inflammatory response syndrome (SIRS) in SFTS patients leads to the overactivation of T-cells and macrophages, causing HLH and clinical disease in patients [63]; the cytokine and chemokine profiles seen in SFTS patients and their potential role in HLH and disease severity are discussed in detail in Section 4.

### 3.5. Animal Models of Tickborne Banyangviruses

Extensive studies have focused on the development of animal models for SFTSV and HRTV in order to gain insights into pathogenesis of these viruses and evaluate medical countermeasures such as therapeutics and vaccines. The generation of a lethal animal model for both SFTSV and HRTV has been restricted by the lack of susceptibility of immunocompetent animals, such as C57/BL6 mice, and hamsters to the infection [64,65,66]. Although infection of C57/BL6 mice with SFTSV demonstrated SFTSV antigen positive-macrophage and platelets in the spleen of infected animals, the clinical signs do not mirror clinical symptoms seen in severe/fatal SFTS patients; infected C57/BL6 mice only exhibited leukopenia at one time point post-infection, which was resolved days later [64]. Susceptibility of animals to either SFTSV or HRTV is achieved when components of the innate immune system are abolished [65,66,67,68,69]. SFTSV caused lethal infection in IFN α/β receptor knockout (IFNAR^−/−^) mice, and signal transduction and activator of transcription 2 knockout (STAT2^−/−^) mice and hamsters [66,68,69]. HRTV infection was lethal in IFN α/β/γ receptor knockout (AG129) mice [67]. Larger animal models such as macaques have been assessed for SFTSV infection as well, however, they do not exhibit all clinical signs seen in SFTS patients [66,70]. An aged-ferret model was demonstrated to be susceptible to lethal SFTSV infection; aged ferrets succumbed with clinical signs of fever, reduction of platelet count and white blood cell count compared to young ferrets [71]. Notably, it was most recently demonstrated that SFTSV infection was lethal in cats and infected cats showed clinical signs such as weight loss, thrombocytopenia, and leukopenia [72]. Upon necropsy of moribund animals, severe gastrointestinal hemorrhage and hemophagocytosis in the bone marrow, lymph nodes, and spleen were observed [72]. Although great strides have been made in the generation of animal models for the study of tickborne banyangvirus infection, further studies examining the role of the immune response following viral infection are needed in order to shed light on pathogenesis of these viruses.

## 4. Host Immune Response to Banyangviruses

Mammalian hosts possess multiple immune barriers that prevent virus to establish successful infection in the host [73]. The IFN response, host innate immunity, is the first line of defense against viral infection that is important for elimination of initial virus replication [73,74]. Pro-inflammatory response also plays a critical role in innate immunity by evoking inflammation, and also aids in the stimulation of adaptive immunity [75]. While the innate immunity is an immediate, non-specific defense against virus infection, the adaptive immune response (humoral and cell-mediated responses) takes days to weeks following infection to generate highly-specific immunity, and also can provide long-lasting protection in the host [76].

Viruses, on the other hand, have also evolved a variety of mechanisms to counteract and/or disrupt host immune system, resulting in the efficient viral replication and severe outcome in the host. IFN antagonism and its molecular mechanism mediated by viral IFN-antagonistic protein have been well-studied in many viruses [77,78]. There are also several reports demonstrated the involvement of viral IFN-antagonistic function in impairment of dendritic cell maturation that disrupts signaling cascade bridging from innate to adaptive immunity [79,80,81,82]. Moreover, clinical studies have been demonstrated that uncontrolled, aberrant pro-inflammatory response following virus infection is associated with disease severity; the systemic inflammatory response syndrome (SIRS) has been observed in severe/fatal diseases, including viral hemorrhagic fevers [82]. Taken together, immune evasion/disturbance by virus significantly contributes to disease severity, and thus investigation of the immune response following infection is critical to not only elucidate pathogenesis but also generate effective vaccines and/or antiviral treatments. In this section, we will focus on host immune responses to tickborne banyangviruses, and discuss about molecular mechanisms of banyangvirus-mediated immune antagonism.

### 4.1. Innate Immune Evasion by Interferon-Antagonistic Function of Banyangvirus NSs

**Figure 3:**
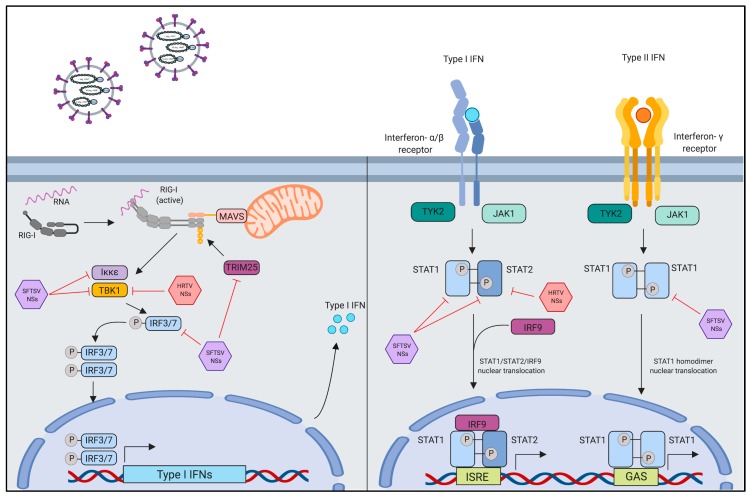
Antagonism of the innate immune response by SFTSV and HRTV. The non-structural proteins of SFTSV and HRTV are responsible for the antagonism of the host interferon response. SFTSV NSs blocks signaling cascades through sequestration of host proteins into viral inclusion bodies; HRTV does not form inclusion bodies, but still interacts and restricts induction of type-I interferons. The SFTSV NSs and HRTV NSs also differentially antagonize the signal component of the interferon response through direct interaction with STAT proteins thereby preventing downstream production of ISGs.

The IFNs, grouped into three subfamilies (type I, II or III), are a group of host secretary proteins that are produced in response to viral infection, playing indispensable roles in eliciting innate antiviral responses [38,83]. The IFN pathway can be explained as two phases, IFN induction and signaling (Figure 3): IFN induction is triggered by pathogen recognition via pattern recognition receptors in the cytoplasm of the infected cells, whereas IFN signaling is activated by binding of secreted IFNs to their cognate receptors expressed on adjacent cells that leads to the expression of antiviral proteins [38]. Important sensing proteins for IFN induction are members of the retinoic inducible gene I (RIG-I)-like family, which trigger signaling cascades following recognition of pathogen associated molecular patterns (PAMPs) [38]. Downstream of RIG-I and the mitochondrial antiviral signaling (MAVS), TANK binding kinase-1 (TBK1) in conjunction with IκB kinase epsilon (Iκκepsilon; Iκκε), mediates phosphorylation of interferon regulatory factor 3 and 7 (IRF3 and IRF7) [38]. Phosphorylation of either IRF3 or IRF7 leads to their homodimerzation and subsequent nuclear translocation for the activation of IFN α/β expression [38]. Type I IFN signaling in virus infection occurs following IFN-α, IFN-β, or IFN-γ binding to their receptors and signaling through the signal transduction and activator of transcription (STAT) proteins [38]. STAT1 and STAT2 heterodimers are important for Type I IFN signaling while STAT1 homodimers play a key role in Type II IFN signaling [84]. Nuclear translocation of STAT homo or heterodimers, with other transcription factors, results in transcriptional activation of IFN-stimulated genes (ISGs) leading to antiviral state in the host [38,84].

It has been well-demonstrated that viruses have evolved to encode their own IFN-antagonistic proteins to counteract both the IFN induction and signaling steps [77,78]: the VP35 protein from Ebola virus antagonizes the IFN response by inhibiting activation of RIG-I through its interaction with the protein activator of the interferon-induced protein kinase (PACT), a cellular protein which can promote RIG-I activation [85,86]. The IFN antagonism mediated by NS4B protein of the flaviviruses, such as West Nile virus and dengue virus, is achieved by blocking TBK1 activation [87]. The V proteins from several paramyxoviruses antagonize the IFN signaling through binding with STAT2 protein [88,89,90,91,92,93,94,95,96]. Within the *Phenuiviridae* family, the IFN antagonistic functions of the non-structural (NSs) proteins of Rift Valley Fever Virus (RVFV) and Uukuniemi virus (UUKV) have been extensively studied and reviewed [97,98,99]. RVFV NSs localizes to the nucleus where it directly inhibits IFN-β promoter activity, and directly prevents the export of host mRNA to the cytoplasm [100,101]. UUKV has not been shown to cause disease in humans; UUKV NSs has weak IFN antagonism activity through its interaction with MAVS [98,99].

Previous reports showed that SFTSV NSs binds and sequesters several host proteins that are important for type I IFN induction, such as TBK1, Iκκepsilon (Iκκε), IRF-3, IRF-7, and tripartite motif 25 (TRIM25), into the NSs-induced “inclusion bodies” or “viroplasm-like structures” [102,103,104,105,106]; the “inclusion bodies” or “viroplasm-like structures” is observed in both SFTSV-infected cells and SFTSV NSs-expressed cells (Figure 3) [104]. The formation of these structures induced by the NSs in SFTSV-infected cells play two roles: (1) formation of viral replication factories, and (2) spatial re-distribution of host proteins important for the type I IFN response [104]. The SFTSV NSs also inhibits type I IFN signaling pathway by interacting with STAT1 and STAT2 heterodimers, inhibiting their phosphorylation status, and nuclear translocation, which leads to suppression of ISGs expression [99,107,108,109,110,111]. Another study implicated the role of the SFTSV NSs in the type II IFN response by demonstrating that SFTSV NSs prevents STAT1 homodimerization, and reduces STAT1 protein level overall thereby reducing IFN-γ production [112]. HRTV NSs, similar to SFTSV NSs, has also been shown to interact with TBK-1 and STAT2 [113,114]. It is hypothesized that HRTV NSs binds STAT2 to inhibit its phosphorylation and subsequent dimerization with STAT1 prior to their nuclear translocation [99,114]. Protein mapping of the SFTSV and HRTV NSs have demonstrated the N-terminal domain, which contains two conserved amino acids position at 21 and 23 was found to be essential for both SFTSV and HRTV NSs to interact with TBK1; binding of the SFTSV NSs prevents TBK1 autophosphorylation and downstream type I IFN activity [105]. Although SFTSV NSs has been reported to sequester several host proteins required for IFN induction and signaling in NSs-induced “inclusion bodies”, it was also shown that forming the inclusion bodies is dispensable for SFTSV NSs-mediated IFN antagonism [105]. Furthermore, two mutations in a PxxP motif in the SFTSV NSs protein that are important for the formation of inclusion bodies reduced, but did not completely abolish IFN antagonism activity of the NSs [102]. Indeed, HRTV NSs also binds to host proteins, but does not form inclusion bodies [99,113,114]. Taken together, role of the inclusion bodies during banyangvirus infection remains to be further investigated.

Several groups have demonstrated the importance of IFN antagonism in banyangvirus infection by the use of animal models. Interestingly, whereas SFTSV does not cause lethal infection in wild-type mice and hamsters, STAT2 knockout mice and hamsters become susceptible to SFTSV infection; this is likely explained by the fact that SFTSV NSs cannot prevent phosphorylation of mouse or hamster STAT2 due to its lack of ability to bind STAT2 from those species [110]. AG129 mice, which lack IFN α/β/γ receptors are susceptible to lethal HRTV infection, while Syrian golden hamsters lacking STAT2 infected with HRTV show clinical signs of disease such as weight loss, and liver lesions but are not fully-susceptible to fatal HRTV infection [65,67].

GTV NSs, much like SFTSV and HRTV NSs, has been reported to antagonize IFN-β promoter activity and expression of ISGs [6]. Similar to SFTSV NSs, GTV NSs also forms inclusion bodies [6]. Subsequent studies are required to fully characterize the IFN-antagonistic capabilities of the GTV NSs.

### 4.2. Pro-Inflammatory Activation and Suppression by Banyangvirus NSs

As well as the IFN system, the host pro-inflammatory response is required in innate immunity and aids in the stimulation of adaptive immunity subsequent virus clearance. This pro-inflammatory response must be balanced with the induction of an anti-inflammatory response, since the excessive pro-inflammatory activation can be a critical contributor to disease severity [82]. Uncontrolled pro-inflammatory response characterized by aberrant production of pro-inflammatory cytokines/chemokines, has been observed in bacteria sepsis and the infection of Ebola virus, Crimean Congo hemorrhagic fever virus, and influenza virus [82,115]. Tumor Necrosis Factor-α (TNF-α), Interleukin-1β (IL-1β), Interleukin-8 (IL-8, CXCL8), and monocyte chemoattractant protein-1 (MCP-1) are notably elevated during the early phases of infection in the case for sepsis, while IL-6 gradually increases over time [116]. The elevated level of IL-10, anti-inflammatory cytokine, is also observed in patient serum following the production of pro-inflammatory cytokines [116]. Systemic inflammatory response syndrome (SIRS) coupled with mixed/compensatory anti-inflammatory response syndrome (MARS/CARS) can result in tissue damage, coagulation abnormalities, and vascular leakage leading to hemorrhage, eventual multi-organ failure, and fatality [117,118].

Much like other viruses causing hemorrhagic fever, SFTSV and HRTV infection likely cause an aberrant pro-inflammatory response through production of pro-inflammatory cytokines and chemokines: the elevated cytokine and chemokine levels during SFTS infection has been well-described, and is hypothesized to correlate to disease severity and HLH as discussed in Section 3.4 [57,58,62,119]. Both fatal and surviving patients have elevated cytokine and chemokine levels during the acute phase of infection, which returns to normal levels in surviving patients at the end of the first week [63]. SFTS patients have increased concentration of TNF-α, IFN-γ, Interleukin-1 (IL-1) receptor agonist (IL1-RA), IFN-α, granulocyte-colony stimulating factor (G-CSF), Interleukin-6 (IL-6), Interleukin-10 (IL-10), IFN-γ-inducible protein-10 (IP-10) with decreased IFN-β, RANTES, platelet derived growth factor-BB (PDGF-BB), and toll-like receptor-3 (TLR-3) expression compared to healthy controls; IL-6, G-CSF, IFN-γ, and MIP-1α were significantly higher in severe SFTS patients compared to mild SFTS patients [63,119]. Furthermore, a separate study identified a unique pattern of the cytokines and chemokines IL-1β, IL-8, MIP-1α, MIP-1β, in fatal SFTS patients, but not surviving patients or healthy controls [120].

The nuclear factor-kappa-light-chain enhancer of activated B cells (NF-κB) is a major transcription factor complex involved in driving inflammation. The activation of canonical NF-κB pathway is represented by phosphorylation and nuclear translocation of NF-κB subunit complex, p65 (RelA)/p50, followed by proteasomal degradation of the inhibitor of κB-α (IκBα), leading to the expression of their target pro-inflammatory genes. Previous reports have been shown the various mechanisms mediated viruses to evade or activate the inflammatory response via NF-κB pathway. Indeed, vaccinia virus, West Nile virus, human immunodeficiency virus, and herpes simplex virus prevent NF-κB signaling by encoding different antagonists that block at various steps of the pathway [121]. On the other hand, influenza A virus has been reported to be dependent on active NF-κB signaling for active viral replication [122]. Rabies virus disturbs stability of TPL2/p105/ABIN2 complex via binding to another host interaction partner participating in this complex, RelAp43 (spliced variant of RelA), leading to the suppression of target gene expression [123,124]. In addition to the role of the banyangvirus NSs protein in evasion of the innate immune response, SFTSV NSs is considered to be involved in the induction of the pro-inflammatory response via activation of NF-κB [46,125]. One group demonstrated transient NF-κB activation during SFTSV infection in human leukemia monocytic cell line, THP-1 [46]. Moreover, it was also shown that the expression of SFTSV NSs induced NF-κB promoter activity in the liver hepatocellular carcinoma cell line, HepG2 [125]. In contrast, studies using HeLa cells derived from human cervical cancer and in 293T, derived from human embryonic kidney cells, showed inhibition of NF-κB activity by SFTSV NSs suggesting that NF-κB activation by SFTSV NSs might be cell-type or tissue specific [125]. Strikingly, it has been shown that pre-treatment of THP-1 cells with an NF-κB inhibitor, 6-amino-4-(phenoxyphenyl-ethylamino)quinozoline, prior to infection with SFTSV, reduced viral titers [46]. Moreover, pre-treatment of liver hepatocellular carcinoma cell line, HepG2, with the NF-κB inhibitor, BAY 11-7082, followed by SFTSV infection led to the reduction of not only pro-inflammatory cytokines and chemokines such as IL-6, IL-8, IP-10, and RANTES, but also viral S gene copy numbers [125]. These insights suggest that inflammatory response induced via NF-κB activation by NSs might enhance viral replication in specific cell types and tissues.

**Figure 4:**
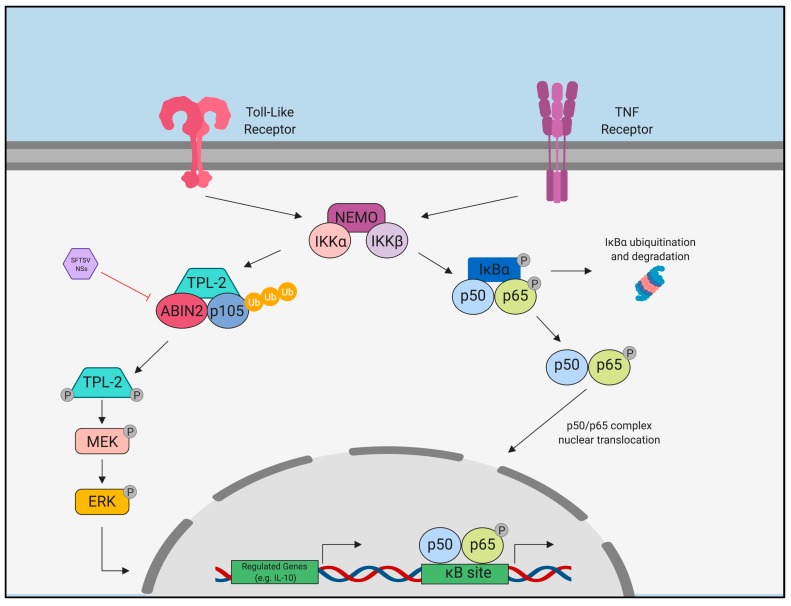
Induction of the TPL2 signaling pathway by SFTSV NSs. The SFTSV NSs binds ABIN2, an inhibitor of TPL-2 and p105, which frees the complex to induce downstream MEK/ERK signaling and production of regulated genes, such as IL-10.

Despite reports indicating that the SFTSV NSs may induce NF-κB-mediated pro-inflammatory response, there are also some reports demonstrating the suppression of inflammatory response by SFTSV infection or expression of SFTSV NSs [46,47]. Microarray and pathway analysis of SFTSV-infected THP-1 monocytes showed an upregulation of several host proteins that inhibit activation of NF-κB subunit complex, p50/p65 and the p52/RelB, indicating suppression of the NF-κB response by SFTSV [46]. Moreover, a previous study demonstrated that the SFTSV NSs binds and inhibits A20-binding inhibitor of NF-κB 2 (ABIN2) to form complex with its interaction partners, tumor progression locus 2 (TPL2) and p105, resulted in the marked upregulation of anti-inflammatory cytokine IL-10 (Figure 4) [47]; p105/TPL2 complex regulates the expression of inflammatory genes, including anti-inflammatory gene *IL10*, in which the signaling cascade is inhibited by ABIN2 binding to this complex. Thus, the interaction of SFTSV NSs with ABIN2 turns out upregulation of IL-10 that can be a cause to dampen host defense and promote viral replication. The infection of recombinant SFTSV possessing NSs with a mutation at the amino acid position 102 from proline to alanine (NSs-P_102_A) reduced the expression of IL-10. Moreover, whereas the infection of SFTSV possessing wild-type NSs was lethal in IFNAR^−/−^ mice, recombinant SFTSV possessing NSs-P_102_A rescued 70% of mice, suggesting IL-10 expression might be a key factor that contributes to the pathogenesis of SFTSV [47]. Interestingly, other reports demonstrated that SFTSV or SFTSV NSs induces the expression of micro-RNA (miRNA)-146b, which is known to mediate macrophage differentiation by upregulation of IL-10, resulting in THP-1 monocytes skewing towards an anti-inflammatory M2 phenotype [48]. The upregulation of IL-10 and generation of an M2 phenotype may indeed be beneficial in the context of a viral infection in order to establish persistence as has been described for other viruses [126]. These results suggest that the SFTSV NSs-mediated induction of IL-10 production via the TPL2 pathway leads to a subsequent anti-inflammatory response, likely due to miRNA-146b inducing M2 macrophage phenotypes to an immunosuppressive phenotype at sites of infection, which may significantly contribute to the pathogenesis of SFTSV. The over-production of IL-10 may be an indication of mixed anti-inflammatory response or compensatory anti-inflammatory response syndrome (MARS/CARS), which is production of anti-inflammatory cytokines mixing with the pro-inflammatory mediators leading to severe disease and has been seen during other viral and bacterial infections [127]. Taken together, these reports indicate that the SFTSV NSs may play a key role in mediating both pro-inflammatory activation and suppression, which may contribute to pathogenesis of SFTSV. Thus far, there have been no published reports demonstrating the effects of the HRTV and GTV NSs proteins on the NF-κB-mediated pro-inflammatory response, or immune suppression via the NF-κB pathway.

### 4.3. Humoral Immune Response to Banyangviruses

The humoral arm of the adaptive immune response is driven by B-lymphocytes. Immunoglobulin-producing B-cells, plasmablasts and plasma cells secrete antibodies which can bind and inactivate pathogens, direct the phagocytosis of the antigen-antibody complexes by phagocytic cells, or induce the complement system [76]. During viral infection, B-cell activation is driven with the aid of helper CD4+ T-cells which drive the proliferation and class-switching events that lead to a weak, IgM response. Long-term immunity is established through the production of IgG, which can take several weeks to generate [128]. Importantly, B-cells also play a role in the cell-mediated response, which is described in Section 4.4, through their roles as antigen presenting cells for naïve T-cells [76]. Induction of the humoral response is vital for clearance of viral infection, and long-term immunity driven by memory B-cells in cases of future infections [76].

**Figure 5:**
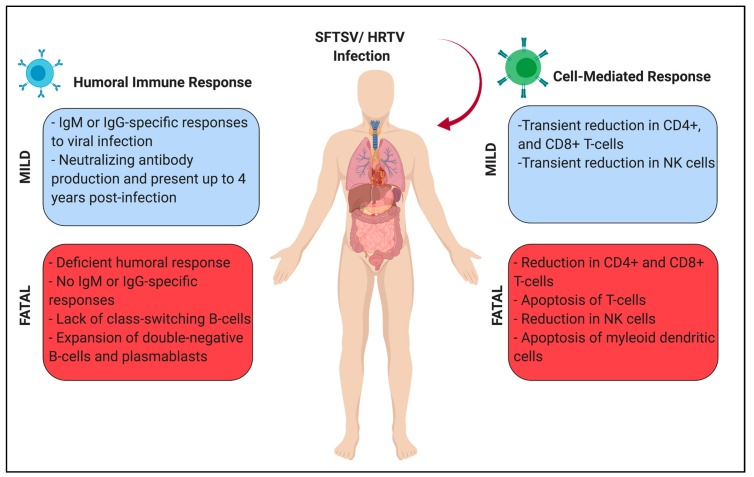
Adaptive Immune Response during SFTSV and HRTV infection. The humoral and cell-mediated responses are described. Fatal patients exhibit a lack of efficient antibody production coupled with the reduction of CD4+ and CD8+ T-cells indicating dysfunction of mounting a robust immune response. Neutralizing antibody responses can be detected years post-hospitalization.

A study of both surviving and deceased SFTS patients in China showed that deceased patients failed to mount either IgM or IgG-specific immune responses for both the SFTSV N and Gn [129]. In contrast, survived patients mounted an IgM-specific response against SFTSV N during the acute phase of infection and developed a Gn-specific IgG response 2–3 weeks post-symptom onset, indicating the humoral immune response is crucial for recovery from SFTS [129]. Investigation of the role of B-cell responses during SFTSV infection demonstrated large B-cell expansion associated with severe SFTS and poor prognosis, while survivors showed a transient increase in B-cells during the mid-phase of infection, which returned to normal level during the convalescent phase [130]; this finding was confirmed by another group indicating a rapid expansion but subsequent exhaustion of B-cells in severe SFTS patients likely contributing to a lack of adaptive response [131]. Importantly, analysis of B-cell subsets from patient cohorts exhibited stark differences between the survived and deceased groups: deceased patients had an increased double negative B-cells (CD27− IgD− cells) and plasmablasts (CD27+ IgD− cells), whereas the proportion of marginal zone B-cells (CD27+ IgD+ cells) in deceased patients reduced compared to survived patients [129]. Additionally, the number of naïve B-cells (CD27− IgD+) in both the survived and deceased group decreases overall, but the total number of naïve B-cells is higher in the survived group [129]. Taken together, these data examining B-cell subsets in SFTS-infected patients indicate dysfunction of B-cell maturation and subsequent failure of humoral immune responses in fatal SFTS patients (Figure 5). Further characterization of plasmablasts from fatal patients exhibited a downregulation of three transcription factors, which are known to be important for class-switching in B-cells: BLIMP-1, IRF-4, and XBP-1 [129]. Thus, these results suggest that abnormalities in B-cell subsets, and failed class-switching in B-cells in patients is a key feature in fatal SFTS-disease. Noteworthy, infection of SFTSV in IFNAR^−/−^ mice, a lethal animal model of SFTSV, followed by histopathological studies demonstrated SFTSV antigens present in immature B-cells in the spleen and lymph node of infected animals [66]. Although there are no current in vitro studies using B-cells from human patients or clinical indications of SFTSV antigen positive B-cells in human patients, B-cells could be a secondary target of SFTSV. Further study is needed to elucidate the role of B-cells in banyangvirus pathogenesis and also in protection from banyangvirus disease.

### 4.4. Cell-Mediated Immune Response to Banyangviruses

The cell mediated immune response is driven by T-cells, such as helper T-cells (CD4+) and effector T-cells (CD8+) [76]: helper T cells regulate/assist in the adaptive immune response via cytokine secretion, while cytotoxic T cells directly kill virus-infected cells by induction of apoptosis. The maturation of naïve T cells is driven via antigen presentation by professional antigen presenting cells, such as dendritic cells, macrophages, and B-cells [76]. These cells are all key players of the cell-mediated arm of the adaptive immune response that must work together in concert in order to establish long-term immunity in the host. Failure of one of several events necessary for each subset of lymphocyte maturation, antigen presentation, or even reduction in overall lymphocyte populations can cause severe disease in the context of a viral infection [76,132,133].

One of the key hematological features found in both surviving and fatal SFTS patients is a lower lymphocyte count compared to healthy controls upon hospital admission. Importantly, CD4+ and CD8+ T-cells from SFTS patients were found to express higher levels of annexin V and Fas/Fas Ligand (FasL), cellular proteins mediating apoptosis, on the cell surface, suggesting the reduction of lymphocytes in patients is due to apoptotic cell death [130,134]. Despite these findings, CD4+ and CD8+ T-cells from surviving patients were also found to proliferate throughout the clinical disease course, and were active as illustrated by high levels of IFN-γ and granzyme B secretion when compared to healthy controls [134]. This indicates that surviving patients are able to rebound following massive lymphocyte apoptosis and mount an immune response to clear SFTSV infection. A previous study showed that poor prognosis SFTS-patients had reduced CD3+, CD4+ and CD8+ T-cell subsets compared to healthy controls and recovery group both at 7–10 days post-symptom onset and at the late stage of infection (>11 days), suggesting the idea that T-cell subsets might be useful to predict prognosis of SFTS patients (Figure 5) [130].

Dendritic cells have been known to be essential for antigen presentation and elicitation of the humoral immune response. However, recent reports indicate that myeloid dendritic cells (mDCs), but not plasmacytoid dendritic cells (pDCs), may also play an important role in SFTS prognosis by aiding in the presentation of antigen to induce an adaptive immune response [135]. Moreover, extensive apoptosis of mDCs from fatal SFTS patients has also been reported, which may play a role in the failure for antigen presentation, and subsequent class-switching for protective immunity combined with dysfunction of T-follicular helper cells [129]. By contrast, in vitro infection of THP-1 monocytes, has not led to induction of apoptosis by SFTSV [46]. These data indicate that the rapid depletion of mDCs may play a key role in the deficient humoral response seen in fatal SFTS patients, and possibly leads to immune paralysis due to the disruption of DC function and DC-T-cell network as observed in Ebola virus disease [80,81]. Moreover, both recovered and fatal SFTS patients had decreased natural killer (NK) cell populations during the later phases of infection at both 7–10 days and >11 days post-symptom onset; NK cell depletion most likely contributes to increased viral load [130]. Taken together, lymphopenia seen in SFTS patients impacts T-cell subsets and NK cell populations, which may hinder the mounting of an immune response and subsequent clearance of the viral infection.

### 4.5. Establishment of Long-Term Immunity

Most recently, a follow-up study of previous SFTS patients identified SFTSV neutralizing antibodies up to four years post-hospitalization [136,137]. While it is not known how long these neutralizing antibodies against SFTSV persist in humans, this indicates the importance of establishment of neutralizing antibody response for recovery from SFTS, and potentially other tickborne banyangviruses. Insights into the immunogens responsible for neutralizing antibodies against tickborne banyangvirus infection have come from generation of vaccines against several banyangvirus proteins. A single dose of recombinant vesicular stomatitis virus (VSV)-based vaccine expressing either SFTSV or HRTV Gn/Gc completely protected immunocompromised mice from lethal SFTSV infection [138], while immunization with SFTSV NSs protein and subsequent SFTSV challenge in C57/BL6 mice did not result in virus clearance [139]. It was also shown that a DNA vaccine encoding only SFTSV Gn/Gc efficiently induces protection against SFTSV infection in an aged ferret model [140]. These results indicate that the viral glycoproteins of tickborne banyangviruses may be important for generation of the humoral response, and long-term protection, however the role of other viral proteins still remain to be elucidated for long-term protection.

## 5. Discussion

Tickborne banyangviruses, specifically SFTSV and HRTV, pose a great threat to human health, and despite their prevalence in eastern Asia and the U.S., respectively, there are no current approved therapeutics or vaccines against these viruses. Furthermore, expanding tick vectors are likely to contribute to cases of SFTSV and HRTV. Although studies assessing the immune cell subsets during SFTS infection and role of the NSs protein in modulating the immune response have been conducted, further studies are required to elucidate the pathogenesis of these significant pathogens causing VHF-like disease.

Innate immune evasion and induction of the pro-inflammatory response likely play dual roles in the pathogenesis of tick-borne banyangviruses. The NSs proteins of these viruses evade the host innate immune response through their IFN-antagonistic functions, which lead to productive viral replication. SFTSV NSs was shown to also induce anti-inflammatory responses mediated by IFN-γ, and IL-10. While SFTSV and HRTV infection drive the induction of high levels of pro-inflammatory cytokines and chemokines which contribute significantly to pathogenesis, this has not been studied extensively in available animal models, nor is it clearly understood what key factors lead to the SIRS induced by tickborne banyangviruses. Further studies are needed to understand the cytokine dynamics over time in patients infected with tickborne banyangviruses.

The impairment of both humoral and cell-mediated immune responses in SFTS fatal patients demonstrate immense immune dysregulation induced by virus infection and likely the immunosuppressive environment leading to lack of antigen presentation and subsequent impairment of class-switching in B-cells. While the studies described in this review shed light on lymphocyte subsets during mild and fatal SFTS infection, further studies are required to elucidate the mechanisms leading to lymphocyte and platelet loss seen in patients, and determine viral proteins responsible for dysregulation of the adaptive response. Moreover, studies in HRTV patients and animal models needs to be conducted to understand these responses, and identify conserved mechanisms among these two genetically related viruses. The clinical impact of GTV remains to be explored. Investigation into the immune responses contributing to VHF-like illness induced by infection of tickborne banyangviruses is a critical area of study for the knowledge of pathogenesis and development of novel therapeutics.

## 6. Conclusions

In this review, we have focused on the impact of tickborne banyangvirus infection on host innate, humoral, and cell-mediated immune responses. While the examination of host immune dysregulation and its mechanism have been mainly conducted in the context of SFTSV infection, further studies will also be required to fully understand the impact of HRTV or GTV in immune responses during infection. Understanding of the molecular mechanisms underlying host immune responses induced by banyangvirus infection will not only lead to the development of novel therapeutics and vaccines against banyangviruses, but also shed light on the mechanisms of immune disturbance during infection of other viruses causing viral-hemorrhagic fever-like illness.
